# Durable Anti-SARS-CoV-2 Antibody Response after mRNA-1273 Booster in Peritoneal Dialysis Patients during the Omicron Wave

**DOI:** 10.3390/vaccines11061121

**Published:** 2023-06-19

**Authors:** Georg Beilhack, Rossella Monteforte, Florian Frommlet, Roman Reindl-Schwaighofer, Robert Strassl, Andreas Vychytil

**Affiliations:** 1 Division of Nephrology and Dialysis, Department of Medicine III, Medical University of Vienna, 1090 Vienna, Austria; georg.beilhack@meduniwien.ac.at (G.B.);; 2 Center for Medical Statistics, Informatics and Intelligent Systems, Medical University of Vienna, 1090 Vienna, Austria; 3 Division of Clinical Virology, Medical University of Vienna, 1090 Vienna, Austria

**Keywords:** peritoneal dialysis, mRNA-1273 vaccine, antibody response, SARS-CoV-2, COVID-19

## Abstract

Anti-SARS-CoV-2 vaccination of dialysis patients has been proven to be safe and effective to reduce COVID-19-related morbidity and mortality. However, data on the durability of anti-SARS-CoV-2 antibodies post-vaccination in peritoneal dialysis (PD) patients are scarce. In this prospective single-center cohort study we measured anti-SARS-CoV-2 RBD antibodies 3 and 6 months after the 3rd dose of the mRNA-1273 vaccine in 27 adult PD patients and recorded breakthrough infections. Furthermore, in a mixed model analysis, we analyzed potential factors influencing the humoral response following vaccination. Anti-SARS-CoV-2 RBD antibody levels declined from 21,424 BAU/mL at 1 month to 8397 BAU/mL at 3 months and to 5120 BAU/mL at 6 months after the 3rd dose, but remained higher than pre-3rd dose levels (212 BAU/mL). Eight patients (29.6%) were infected with SARS-CoV-2 within six months from the 3rd dose during the Omicron wave. Previous high antibody levels, high glomerular filtration rate (GFR) and low Davies Comorbidity Score were associated with higher anti-SARS-CoV-2 antibody levels after the booster. In conclusion, PD patients exhibited a robust and durable humoral response after a third dose of the mRNA-1273 vaccine. A high GFR and low comorbidity as well as previous high antibody levels predicted a better humoral response to vaccination.

## 1. Introduction

Dialysis patients are at high risk for SARS-CoV-2 infection leading to increased morbidity and mortality compared with healthy individuals [1,2]. Known factors that may contribute to the increased susceptibility of dialysis patients to infections include advanced age, premature aging of their immune system, the presence of comorbidities, frailty, uremia, immunosuppressive therapy, longer dialysis vintage, and elevated chances of exposure while receiving treatment at dialysis centers [3]. 

Furthermore, these factors lead to a compromised immune response in dialysis patients, resulting in a diminished efficacy of vaccination. Dialysis patients commonly exhibit a lower rate of seroconversion and experience a more rapid decline in antibody levels following immunization, which are crucial for protecting against infections. Consequently, the effectiveness of vaccination in dialysis patients is uncertain in terms of achieving a satisfactory immune response. An illustrative example of this attenuated immune response is observed in the protocol involving hepatitis B virus vaccination (Engerix B^®^), where the standard regimen yields a diminished seroconversion rate of merely 40–50% among dialysis patients, in stark contrast to the seroconversion rate of up to 90% observed in healthy individuals [4]. Similar observations of a reduced immune response after vaccination are evident in dialysis patients receiving the annually recommended influenza vaccine and those vaccinated against pneumococcal infections. In dialysis patients, the immune response rates following a trivalent influenza vaccine can be as low as 30–40%, in contrast to a response rate of 70% in healthy adults [5]. Similarly, the immune response is diminished following vaccination against pneumococcal capsular polysaccharide when compared with a control group [6].

Vaccination of dialysis patients against SARS-CoV-2 with mRNA-1273 or BNT162b2 vaccines resulted in notably high rates of seroconversion (more than 90% after two doses) among these patients [7]; it was proven to be safe and helped to reduce hospitalization, severe illness, and death [8,9,10,11]. Several studies in dialysis patients have shown that SARS-CoV-2 antibody levels declined after the two-dose vaccination scheme and that a third vaccine dose as a “booster” induced a strong humoral response in both hemodialysis (HD) and peritoneal dialysis (PD) patients [12,13,14,15,16,17,18,19,20,21]. Most of these studies were investigating HD patients vaccinated with BNT162b2, whereas few studies focused on PD patients using mRNA-1273 [22,23,24,25].

Although the third dose led to a robust humoral response immediately after vaccination, data on the durability of anti-SARS-CoV-2 RBD antibodies are scarce. Since higher anti-SARS-CoV-2 RBD antibody levels are associated with increased protection from COVID-19 and its related complications [26,27], it is crucial to elucidate the antibody kinetics over a longer time interval following the booster dose. The aim of this study was to prospectively measure anti-SARS-CoV-2 RBD antibody levels in our PD cohort 3 and 6 months after the 3rd dose of mRNA-1273. Furthermore, we recorded breakthrough infections during the Omicron-variant-dominated period and identified potential factors influencing the humoral response after vaccination. 

## 2. Patients and Methods

### 2.1. Study Population

This prospective single-center cohort study was conducted at the Division of Nephrology and Dialysis, Department of Medicine III, Medical University of Vienna, Austria. The participants in this study were 27 adult PD patients who had received 3 doses of the mRNA-1273 (Spikevax^®^, Moderna) vaccine against SARS-CoV-2. Here, we assessed anti-SARS-CoV-2 RBD antibody levels 3 months and 6 months after the 3rd dose. The details of the vaccination scheme and data on the humoral response after two doses and one month after the third dose have been published recently [22,28]. None of the patients included in this study (*n* = 27) had a COVID-19 history. Patients were followed up for 6 months from the 3rd vaccination for breakthrough SARS-CoV-2 infection. Patient demographic characteristics, including age and sex, the presence of comorbidities (diabetes, vascular disease, glomerulonephritis, adult polycystic kidney disease, Alport syndrome, GVHD-associated thrombotic microangiopathy, cardiorenal syndrome, secondary focal segmental glomerulosclerosis, AL-amyloidosis, glomerulosclerosis, nephronophthisis, scleroderma), dialysis vintage, dialysis adequacy (Kt/V), residual kidney function (GFR), laboratory data, including hematologic and biochemical parameters (hemoglobin, leukocytes, thrombocytes, albumin, sodium, potassium, calcium, phosphate, bicarbonate, C-reactive protein), and concomitant medications (immunosuppressive therapy, RAAS-inhibitors, vitamin D) were collected from the electronic clinical records of our hospital. For the calculation of GFR, patients were asked to collect urine for 24 h. At the end of the collection period, a blood sample was taken. Because of the tubular secretion of creatinine and tubular absorption of urea, GFR was calculated as the average of renal creatinine and urea clearance, as recommended by recent guidelines [29]. We calculated the comorbidity score of our PD patients according to Davies et al [30]. In brief, seven categories of comorbidities (malignancy, ischemic heart disease, peripheral vascular disease, left ventricular dysfunction, diabetes mellitus, systemic collagen vascular disease, and other significant pathologies, such as severe chronic obstructive disease, cirrhosis, and severe psychiatric conditions) were considered to calculate this score, giving a theoretical maximum score of seven points [30]. In our PD patient cohort [28], the maximum Davies Comorbidity Score reached five points at baseline, while the Davies Comorbidity Score for the current study participants reached a maximum of three points.

### 2.2. Peritoneal Dialysis Modalities

Peritoneal dialysis is performed through the instillation of hypertonic dialysis solution into the peritoneal cavity of PD patients. Osmosis and diffusion processes facilitate the exchange of solutes and fluids, addressing impaired renal clearance. In our present cohort of PD patients, the majority undertook automated peritoneal dialysis (APD, represented by 25 patients) while a minority utilized continuous ambulatory peritoneal dialysis (CAPD, represented by 2 patients). While CAPD is a manual method that can be performed in any suitable location, including dialysate exchanges which are usually repeated three to four times daily, APD is a machine-assisted procedure that is typically carried out during the nighttime hours with a cycler.

### 2.3. Serological Assessment

Blood samples were collected at 3 months (from February to March 2022, median 104 days, IQR 96–112 days) and at 6 months (from April to May 2022, median 174 days, IQR 167–188 days) after the 3rd dose. Antibodies against the receptor-binding domain (RBD) of the SARS-CoV-2 spike (S) protein and the nucleocapsid (N) antigen were quantified as previously described [22,28].

### 2.4. Statistical Analysis

Antibody titer values were log-transformed to compensate for right skewness. For the transformed data, mixed model analysis with the patient as a random effect was performed using the R package lme4 and the lmerTest package to calculate *p*-values [31,32]. 

The initial model included the log-transformed titer values at baseline (after the first mRNA-1273 dose) as a covariate. This mixed model was then amended by one additional predictor, including the variables of age, gender, serum albumin, GFR, Davies Comorbidity Score, serum ferritin, and serum vitamin D, respectively. Descriptive statistics are presented as either mean and standard deviation or median and interquartile range (IQR).

Comparisons of antibodies between two groups were analyzed using the Wilcoxon rank sum test (unpaired two-sample analysis) or Wilcoxon signed-rank test (paired samples). 

## 3. Results

**Figure 1:**
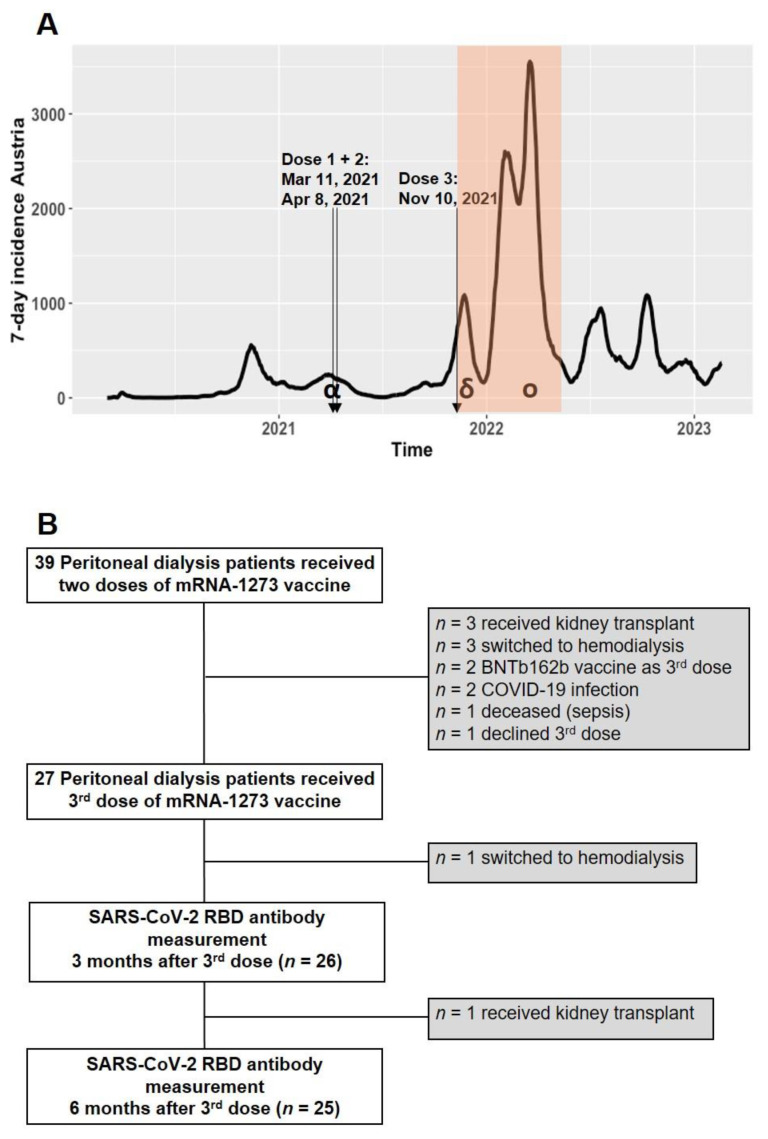
(**A**) Vaccination timeline. PD patients received 3 doses of mRNA-1273 (arrows) and were followed up for 6 months after the 3rd dose (orange segment). The background shows the waves of SARS-CoV-2 infections and dominant variants in Austria (7-day incidence cases per 100,000). (**B**) Flowchart of the peritoneal dialysis patient cohort.

This study is a continuation of our previous published studies [22,28]. The vaccination timeline of included patients and SARS-CoV-2 waves in Austria are illustrated in Figure 1A. Of the original cohort of 39 PD patients, 27 individuals (17 men and 10 women) received a 3rd dose of mRNA-1273, as shown in Figure 1B. We conducted a prospective follow-up study on these 27 PD patients and monitored their anti-SARS-CoV-2 RBD antibody levels for 6 months after receiving the 3rd dose of mRNA-1273 (November 2021 to May 2022). A patient flow diagram and detailed characteristics of the study cohort are shown in Figure 1B and Table 1, respectively.

### 3.1. Anti-SARS-CoV-2 RBD Antibodies Declined within Six Months after the Third Dose of mRNA-1273 in SARS-CoV-2 Naive Patients

**Figure 2:**
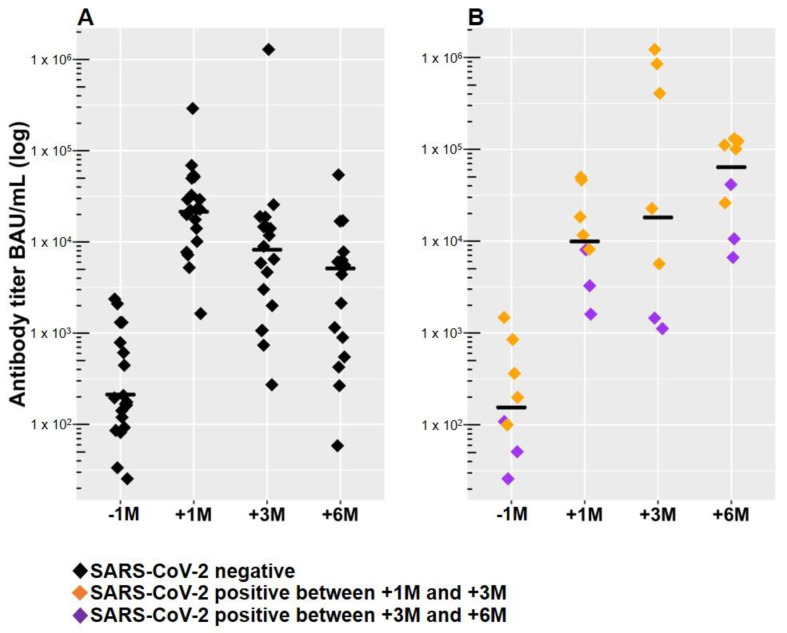
Anti-SARS-CoV-2 RBD antibody levels in SARS-CoV-2-naive (**A**) and SARS-CoV-2 recovered (**B**) PD patients in response to the third mRNA-1273 vaccine. (**A**) Within 6 months after the 3rd dose, antibody levels of corona-naive PD patients declined (6.4-fold change) but did not reach pre-3rd dose levels. (**B**) In contrast, PD patients who were infected by SARS-CoV-2 within the 6 months interval following the 3rd dose had increasing antibody levels after infection (7.4-fold change). Median values are marked by a horizontal line. “−1M” = 1 month before the 3rd dose = 6 months after the 2nd dose; “+1M” = 1 month after the 3rd dose; “+3M” = 3 months after the 3rd dose; “+6M” = 6 months after the 3rd dose; black diamonds—SARS-CoV-2; orange diamonds—SARS-CoV-2 infection between 1 and 3 months after the 3rd dose; violet diamonds—SARS-CoV-2 infection between 3 and 6 months after 3rd dose.

Seventeen patients remained SARS-CoV-2 naive up to six months after the third dose of mRNA-1273. Two patients out of twenty-seven were excluded during the six-month observation time (one patient switched to HD, and one patient received a kidney transplant) (Figure 1B). Anti-SARS-CoV-2 RBD antibodies declined from 21,424 BAU/mL (IQR 11,402–43,650) at 1 month to 8397 BAU/mL (IQR 1867–14,885) at 3 months and to 5120 BAU/mL (IQR 768–7159) at 6 months after the 3rd dose (Figure 2A and Table 2). The median fold-change reduction of anti-SARS-CoV-2 RBD antibody levels was 3.5 between 1 and 3 months and 6.4-fold between 1 and 6 months after the 3rd dose. However, anti-SARS-CoV-2 RBD antibody levels at 6 months after the 3rd dose were significantly higher than those at 6 months after the 2nd dose (5120 BAU/mL vs. 212 BAU/mL, median fold-change 26.7) (Figure 2A). Similarly, SARS-CoV-2-naive patients receiving immunosuppressive treatment (*n* = 4) had declining antibody levels, albeit at 6 months post-3rd dose, they displayed higher levels than at 6 months after the 2nd dose (median 6173 BAU/mL vs. 566 BAU/mL, median fold-change 10.9).

### 3.2. Breakthrough Infections in PD Patients within Six Months after the Third Dose of mRNA-1273

A total of 8 out of 27 PD patients (29.6%) were infected with SARS-CoV-2 within 6 months from the 3rd dose. Time to infection ranged from 69 to 156 days after the 3rd dose. These infections occurred between January and May 2022 during the Omicron BA.1/BA.2 wave in Austria. All patients experienced none (*n* = 3) or mild symptoms (*n* = 5) without hospitalization and with favorable clinical outcomes (Table 3). 

Before SARS-CoV-2 infections occurred, these patients had antibody levels that were 2.16 times lower compared with PD patients that remained SARS-CoV-2 naive (one month after the third dose: 9927 BAU/mL vs. 21,424 BAU/mL, *p* = 0.19, Wilcoxon rank sum test). Anti-SARS-CoV-2 RBD antibodies significantly increased in patients with breakthrough infections from 9927 BAU/mL (IQR 8222–22,586) at 1 month to 18,094 BAU/mL (IQR 3677–769,500) at 3 months and to 73,500 BAU/mL (IQR 23,815–111,825) at 6 months after the 3rd dose (Figure 2B and Table 2). Six months after the third dose, anti-SARS-CoV-2 RBD antibody levels in SARS-CoV-2 recovered patients were significantly higher than in SARS-CoV-2 naive patients (*p* = 0.0003, Wilcoxon rank sum test). 

### 3.3. Previous Antibody Levels, High GFR, and Low Davies Comorbidity Score Are Predictors of a Robust Anti-SARS-CoV-2 Antibody Response

In this study, we performed a mixed model analysis for repeated measurements to investigate potential factors associated with anti-SARS-CoV-2 RBD levels after vaccination with mRNA-1273. The following predictor variables were considered: age, gender, serum-albumin, GFR, Davies Comorbidity Score, serum ferritin, serum vitamin D, and the level of SARS-CoV-2 RBD antibodies after the first mRNA-1273 dose (baseline level). 

The most significant predictor for a robust antibody response after the second and the third dose was the level of anti-SARS-CoV-2 RBD antibodies at baseline (*p* = 1.953 × 10^−11^). Patients with higher antibody levels at baseline showed higher antibody levels after the second and third doses. Additionally, a higher GFR and a lower Davies Comorbidity Score were statistically significant factors (*p* = 0.047 and *p* = 0.034, respectively) associated with higher SARS-CoV-2 RBD antibodies (Table 4). While the effect of the Davies Comorbidity Score on antibody levels was consistent at all time points, GFR had a stronger effect on antibody levels after the first and second doses, which declined after the third dose. This declining effect of GFR on antibody levels could be due to the constant loss of GFR over time in dialysis patients. In our PD cohort that received the 3rd mRNA-1273 dose, the median GFR was 3.18 mL/min (IQR 1.0–6.0) at baseline and 1.86 mL/min (IQR 0.65–4.69) before the 3rd dose (*p* = 0.0006, Wilcoxon signed-rank test).

## 4. Discussion

Our study shows that SARS-CoV-2 RBD antibody levels decline within 6 months after the 3rd dose of mRNA-1273 in SARS-CoV-2 naive PD patients. Nevertheless, they remain higher compared with levels 6 months after the 2nd dose. Our findings are in line with 2 recent studies in HD and PD patients using the BNT162b2 vaccine, in which antibody levels decreased over 6 months after the 3rd dose but remained higher than the pre-3rd dose levels [17,24].

During the 6 months follow-up after the 3rd mRNA-1273 dose, Omicron BA.1 and BA.2 emerged as the predominant variants of concern in Austria. Until 1 month after the third dose, all PD patients included in this study were SARS-CoV-2 naive. In the time interval between 2 and 6 months after the 3rd dose, approximately 30% of the patients were infected with SARS-CoV-2. Prior to SARS-CoV-2 infection, these PD patients had a lower antibody level compared with SARS-CoV-2 naive patients, but this difference did not reach statistical significance, probably due to the small number of patients in our cohort. This inverse relationship between antibody levels and risk for breakthrough infections has been widely reported [26,27,33,34]. In a study by Manley et al. in 16,213 dialysis patients during the pre-Delta and Delta-variant-dominated periods, lower anti-spike IgG levels were associated with a higher risk for COVID-19 infections and COVID-19-related hospitalizations [26]. Furthermore, during the Omicron-dominated period, Montez-Rath et al. observed in 3576 dialysis patients a higher risk of infection among patients with lower circulating RBD IgG [27].

After infection, SARS-CoV-2 RBD antibody levels increased and were higher than those in SARS-CoV-2 naive patients at 6 months after the 3rd dose. Accordingly, several studies in HD and PD patients described a significant increase in SARS-CoV-2 RBD antibodies after infection [24,35]. Quiroga et al reported that high antibody levels after SARS-CoV-2 infection could lead to an enhanced humoral response comparable to a subsequent booster dose [33]. 

In our cohort, the strongest predictor of robust and durable antibody response after the third dose was a high level of anti-SARS-CoV-2 RBD antibodies after the first dose. This correlation between a substantial humoral response after the third dose and high antibody levels after previous doses is supported by other studies [17,25]. On the contrary, Bensouna et al. described that PD and HD patients with high anti-S1 antibodies after two doses did not profit from a third dose with regard to an increase in antibody levels [14]. A possible explanation for this difference could be the vaccine type (BNT162b2) and the shorter intervals between vaccine doses used by Bensouna et al. Among the other factors included in our mixed model analysis, a lower Davies Comorbidity Score and a higher GFR were associated with higher SARS-CoV-2 RBD antibody levels. 

GFR serves as a surrogate parameter for residual renal function, with a higher GFR signifying a better residual renal function. It is known that clinical outcomes for dialysis patients are influenced by the state of their residual renal function. This is due to a range of mechanisms, such as improved solute clearance, effective elimination of uremic toxins, and control of electrolytes, all of which have beneficial effects on inflammation, anemia, diabetes mellitus, and cardiovascular disease [36]. Moreover, an enhanced residual renal function (or higher GFR) affects the adaptive and innate immune responses, primarily through the removal of uremic toxins [37].

Our analysis showed that the effect of the Davies Comorbidity Score was consistent at all time points, while the effect of GFR weakened after the third vaccine dose, probably due to a declining GFR over time. In accordance with our findings, other studies reported that comorbidities and impaired kidney function have a negative effect on the antibody response to SARS-CoV-2 vaccination [3,14,38]. Reduced residual renal function and the presence of comorbidities have been shown to be associated with an impaired immune response to other vaccinations [39,40,41]. For dialysis patients, several mechanisms may contribute to this decreased immune response. These mechanisms include impaired pattern recognition, hyporeactive monocytes, impaired activation of T lymphocytes, and decreased B cell counts due to uremic toxins [37]. Additional factors have been described in HD and PD patients to have a beneficial impact on the humoral response after the third vaccine dose, including younger age and absence of immunosuppressive therapy [14,17,42,43]. Immunosuppressive therapy is associated with a reduced humoral response to anti-SARS-CoV-2 vaccination in HD patients [14,42]. However, in our cohort, PD patients with and without immunosuppressive therapy showed a comparable intensity and durability of the humoral response to the third dose. This observation is in contrast to other studies and could be explained by the limited number of patients with immunosuppressive therapy in our study, the different type and dose of immunosuppressive therapy and vaccine as well as the vaccination scheme used.

Our study presents certain limitations. Our cohort consisted of a rather limited number of PD patients due to the single-center study design, which restricted our ability to perform sub-analyses, such as identifying risk factors for breakthrough infections. Despite the small sample size, our PD cohort was very well characterized, received exclusively mRNA-1273 for all three doses at the same time points, and was SARS-CoV-2 naive until approximately two months after the third dose. Another limitation of our study was the lack of a control group, which prevented a comparison between PD patients and healthy subjects. Only a few studies have compared the response to the third dose of mRNA vaccine between these two groups. Biedunkiewicz et al compared 10 PD patients and 20 healthy controls and found that both groups had a similar waning pattern after the 2nd mRNA vaccine dose and a good response to the 3rd dose [23]. However, PD patients had significantly lower anti-SARS-CoV-2 levels compared with healthy controls. Other studies have included PD patients and healthy subjects as the control group, although differences in vaccine type (non-mRNA vaccine or BNT162b2 instead of mRNA-1273), the different number of vaccine doses between PD patients and healthy controls, the lack of anti-nucleocapsid antibody measurements, and the absence of a third dose in both groups make a comparison with our study difficult [20,44,45]. In addition, our measurements were limited to anti-SARS-CoV-2 RBD antibodies and did not include neutralizing antibodies or assessments of cellular responses to vaccination. However, recent data indicate that both neutralizing and anti-SARS-CoV-2 RBD antibodies correlate with vaccine efficacy and that anti-SARS-CoV-2 RBD antibodies could be used as a surrogate for neutralizing antibody assays [46,47,48,49,50]. 

## 5. Conclusions

In this prospective longitudinal study, we show a strong and durable humoral response in PD patients after a third dose of the mRNA-1273 vaccine. Six months after the third dose anti-SARS-CoV-2 RBD antibodies declined but remained higher than pre-third dose antibody levels. A high GFR and low Davies Comorbidity Score as well as previous high antibody levels predicted a better humoral response to vaccination. In the light of newly emerging SARS-CoV-2 variants of concern as well as the unknown threshold of antibody levels providing durable and robust protection from severe COVID-19, it might be important to monitor antibody levels after vaccination in this vulnerable group of patients since high anti-SARS-CoV-2 antibody levels correlate with vaccine efficacy, lower rates of breakthrough infections, and better clinical outcomes.

## Figures and Tables

**Table 1:** Characteristics of PD patients who received a third dose of mRNA-1273 vaccine.

Patient Demographics	Total (*n* = 27)
Age (years, mean, range)	54.9 (33–76)
Men (%)	17 (63)
Davies Comorbidity Score (%)	
0	11 (41)
1	9 (33)
2	5 (19)
3	2 (7)
4	0
5	0
Dialysis vintage, months median (IQR)	21.5 (13.4–38.7)
Weekly total (renal + peritoneal) Kt/V median (IQR)	1.94 (1.76–2.10)
GFR (mL/min) median (IQR)	1.86 (0.65–4.67)
Laboratory (mean ± SD)	
Hemoglobin (g/dL)	10.4 ± 1.3
Leukocytes (G/L)	7.66 ± 2.19
Thrombocytes (G/L)	230 ± 76.5
Albumin (g/L)	36.3 ± 4.37
Sodium (mmol/L)	135 ± 3.65
Potassium (mmol/L)	4.28 ± 0.51
Calcium (mmol/L)	2.24 ± 0.21
Phosphate (mmol/L)	1.93 ± 0.44
Bicarbonate (mmol/L)	26.6 ± 2.76
C-reactive protein (mg/dL)	0.78 ± 1.02
Medication *n*, (%)	
Immunosuppressive therapy	5 (18.5)
RAAS-inhibitor medication	14 (51.8)
Vitamin D medication	18 (66.7)

**Table 2:** Anti-SARS-CoV-2 RBD antibody levels after mRNA-1273 vaccination in PD patients.

	Antibody Titer (BAU/mL)		
	Total Population (*n* = 27)	SARS-CoV-2 Naive Patients (*n* = 19)	SARS-CoV-2 Infected Patients (*n* = 8) *
6 months after 2nd dose	185 (88–757)	212 (99–783)	156 (83–542)
1 month after 3rd dose	19,405 (8884–40,650)	21,424 (11,402–43,650)	9927 (8222–22,586)
6 months after 3rd dose	6832 (1839–27,690)	5120 (768–7159)	73,500 (23,815–111,825)

* SARS-CoV-2 infection occurred between 2 and 6 months after the 3rd mRNA-1273 dose.

**Table 3:** Characteristics of PD patients with breakthrough SARS-CoV-2 infection after 3rd dose of mRNA-1273 vaccine.

Age (Years)	Sex	Days between 3rd Dose and Infection	Clinical Symptoms	Outcome	Antibody Titer (Median, BAU/mL)	
					1 Month after 3rd Dose	6 Months after 3rd Dose
54	M	69	Mild	Favorable	9927	109,800
33	F	82	Mild	Favorable	51,900	124,200
59	M	86	Mild	Favorable	9620	116,100
43	M	92	Mild	Favorable	16,214	110,400
35	F	109	Mild	Favorable	41,700	27,690
38	F	125	None	Favorable	1820	37,200
56	M	130	None	Favorable	9927	12,190
55	M	156	None	Favorable	4029	6832

**Table 4:** Effects of covariates on anti-SARS-CoV-2 RBD antibody levels after mRNA-1273 vaccine.

	Coefficient	F Value	*p*-Value
Anti-SARS-CoV-2 RBD levels after 1st dose	1.67	87.20	**1.95 × 10^−11^**
Davies Comorbidity Score	−0.48	4.87	**0.034**
GFR	0.13	4.25	**0.047**
Age	−0.03	2.47	0.12
Gender (male)	−0.61	1.28	0.27
Ferritin	0.0005	0.61	0.44
Albumin	0.107	2.12	0.15
Vitamin D	−0.02	3.91	0.055

GFR, glomerular filtration rate.

## Data Availability

The data presented in this study are available upon request from the corresponding author.

## Abbreviations

SARS-CoV-2 severe acute respiratory syndrome coronavirus 2; coronavirus disease 2019 (COVID-19); HD hemodialysis; PD peritoneal dialysis; BAU binding antibody units.
